# A Baseline Assessment of National Health Program Quality in Urban Primary Healthcare Centers in Berhampur, Odisha

**DOI:** 10.7759/cureus.86332

**Published:** 2025-06-19

**Authors:** Madhumita Bhakta, Durga M Satapathy, Jasmin N Panda, Pramila Marandi, Swamy S.V.N., Trupti Das, Abhimanyu Behera

**Affiliations:** 1 Community Medicine, Maharaja Krushna Chandra Gajapati (MKCG) Medical College and Hospital, Berhampur, IND

**Keywords:** national health programs, observational study, primary healthcare, quality assessment in healthcare, urban health

## Abstract

Introduction: Quality healthcare underpins effective service delivery and positive health outcomes, yet many health systems worldwide grapple with infrastructure deficits and inequitable access. In India, urban primary healthcare centers (UPHCs) frequently struggle to deliver consistent quality, and Berhampur, Odisha, is no exception: workforce shortages and limited health infrastructure continue despite government initiatives. A thorough baseline assessment of the quality of National Health Programs (NHPs) in Berhampur’s UPHCs is therefore essential to identify service gaps, strengthen accountability, and inform targeted improvements aligned with national quality assurance standards.

Methodology: This observational study, conducted from September 2023 to February 2024 across all eight UPHCs within the Berhampur Municipal Corporation, employed the NHP component of the National Quality Assurance Standards (NQAS) checklist to evaluate each facility’s performance. Universal sampling ensured comprehensive coverage, and compliance with measurable checklist elements and checkpoints was recorded. Departmental scores were calculated using formulated Excel Sheets by summing compliant items, converting these to percentages, and displaying results via a visual scorecard to facilitate interdepartmental comparisons.

Results: Performance varied markedly: Ankuli scored 28.52%, Aska Road 93.16%, Aga Sahi 94.49%, Ambapua 91.25%, Baikuntha Nagar 92.21%, Goodshed Road 86.50%, Khodasingh 89.54%, and Uttaramukhi 89.54%. While several PHCs demonstrated high adherence to NHP standards, Ankuli’s low score highlights critical deficiencies requiring urgent attention.

Conclusions: The study concludes that national-quality assessments demand extensive preparatory work-including training external assessors, sensitizing state nodal officers and facility staff, and ensuring widespread familiarity with program protocols and frameworks to achieve sustainable, long-term improvements in healthcare delivery.

## Introduction

Quality in healthcare is a fundamental measure of the scale and range of elements of care provided, encompassing both individual and population-based targets [1]. Quality assurance in healthcare necessitates the conversion of healthcare processes into measurable health outcomes [2]. The assessment of the baseline status of the Quality of National Health Programs (NHPs) ongoing in the urban primary healthcare centers (UPHCs) of Berhampur is essential for ensuring effective healthcare delivery and improving health outcomes among the urban population [3]. According to global statistics, ensuring quality healthcare remains a challenge worldwide. The World Health Organization (WHO) reports that many countries face significant barriers to achieving high-quality healthcare, resulting in adverse health outcomes and disparities in access to care [4]. In India, the quality of healthcare services varies widely across different regions and healthcare facilities, with rural areas often facing significant challenges in terms of infrastructure, resources, and service delivery [5].

Furthermore, in the state of Odisha, especially in Berhampur, the healthcare system faces numerous challenges, including limited infrastructure, healthcare workforce shortages, and disparities in access to healthcare services [6]. Despite government initiatives to improve healthcare quality, gaps in implementation and adherence to quality standards persist, particularly in urban primary healthcare settings [7].

Therefore, this descriptive observational study aims to assess the baseline status of the Quality of NHPs ongoing in the UPHCs of Berhampur. The findings of this study will inform targeted interventions and policy measures aimed at enhancing the quality of healthcare services and improving health outcomes for urban populations in Berhampur, Odisha.

## Materials and methods

This study adopted an observational cross-sectional design to evaluate the baseline status of the quality of National Health Programs (NHPs) implemented at UPHCs operating under the administrative jurisdiction of the Berhampur Municipal Corporation, located in Ganjam district, Odisha, India. The study was conducted over a period of six months, from September 2023 to February 2024. This duration allowed adequate time for planning, data collection, validation, and analysis of the service quality across all designated PHCs. The study population consisted of all eight UPHCs functioning under the municipal corporation, namely: Ankuli, Aska Road, Ambapua, Aga Sahi, Baikunthanagar, Goodshed Road, Khodasingh, and Uttaramukhi. A universal sampling technique was employed to include all eligible PHCs in the evaluation, ensuring comprehensive coverage and representativeness of the urban health service landscape within the municipal area. Inclusion criteria encompassed all UPHCs that were fully operational and providing services under the umbrella of various NHPs during the study period. Conversely, PHCs that were under renovation, temporarily closed, or non-functional during the data collection window were excluded to avoid bias due to incomplete or unavailable service data.

The data collection process was conducted using the National Quality Assurance Standards (NQAS) checklist, specifically the component related to the assessment of NHPs. The NQAS checklist is a standardized, government-approved tool developed by the Ministry of Health and Family Welfare (MoHFW), Government of India, for assessing and benchmarking the quality of services delivered in public health facilities. This tool includes predefined standards, measurable elements, and checkpoints covering various thematic areas and operational components of health program implementation. The NQAS checklist, while standardized nationally, was minimally adapted to suit the local context during the data collection process. Minor contextual modifications included translating selected checklist items into the regional language to aid comprehension by facility staff and emphasizing specific health programs more prominently based on their local relevance and prevalence (e.g., prioritizing vector-borne disease control in endemic areas). Additionally, certain assessment criteria were clarified through locally relevant examples to ensure uniform understanding among assessors. However, the core structure, standards, and measurable elements of the original MoHFW-approved checklist were retained without alteration to preserve its validity and comparability.

Trained investigators conducted onsite evaluations of each PHC using this checklist. During the assessment, the level of compliance with each checkpoint was scored as follows:

(1) 2 points for full compliance (100%)

(2) 1 point for partial compliance (50%-99%)

(3) 0 points for non-compliance (<50%)

Each PHC’s scores were recorded manually and then entered into a customized Microsoft Excel spreadsheet. The raw scores were aggregated, and the final departmental score for each service area was calculated as a percentage of the total possible score, allowing for standardized comparison. These scores were then compiled into a departmental scorecard, which provided a visual and tabular representation of performance across various areas of concern, facilitating inter-facility and inter-departmental comparisons.

The data underwent statistical analysis using Microsoft Excel for preliminary computations and Jamovi software (version 2.3.66) for advanced statistical treatment and graphical presentation of results. Descriptive statistics such as means, percentages, and standard deviations were used to summarize and interpret the data. The results were reported in the form of tables and figures, offering both quantitative and visual insights into the quality gaps and strengths across the UPHCs.

To ensure data quality and validity, standardized procedures were implemented for all assessments, and periodic cross-verification of the scoring was undertaken by senior members of the research team to eliminate inconsistencies or observer bias. The study was conducted following ethical principles. Ethical clearance was obtained from the Institutional Ethics Committee before data collection (vide approval number 1334). All investigators adhered to strict ethical guidelines concerning participants' confidentiality, data protection, and nonmaleficence, as no personal identifiers or patient-level data were collected during the facility assessments.

## Results

The assessment of the baseline status of the quality of NHPs, ongoing in the PHCs of Berhampur, revealed varying levels of compliance with the NQAS across different facilities (Table 1).

**Table 1:** Facility-wise NHP performance scores in percentage. The table displays the percentage scores of various urban primary health centers (UPHCs) based on the assessment of the National Health Program (NHP) implementation. The scores reflect the level of compliance and performance of each facility in relation to national program standards and indicators.

Facility	NHP score (%)
Ankuli	28.52
Aska Road	93.16
Aga Shahi	94.49
Ambapua	91.25
Baikunthanagar	92.21
Goodshed Road	86.50
Khodasingh	89.54
Uttaramukhi	89.54

Among the PHCs assessed, Aska Road, Aga Shahi, Ambapua, and Baikunthanagar demonstrated high levels of compliance, achieving impressive NHP scores of 93.16%, 94.49%, 91.25%, and 92.21%, respectively. Goodshed Road, Khodasingh, and Uttaramukhi PHCs also showed satisfactory adherence to NQAS standards, obtaining respectable scores of 86.50%, 89.54%, and 89.54%, respectively. In contrast, Ankuli PHC obtained a lower NHP score of 28.52%, indicating areas for improvement in compliance with NQAS standards.

Across the eight service domains (Table 2), mean compliance with NQAS ranged from a high of 93.8% in Quality Management to a low of 61.7% in Outcome, with overall mean scores clustering above 80% except for Patients’ rights (mean 75.9%) and Outcome. Medians (78.6%-100%) exceeded means in most domains, indicating left-skewed distributions driven by low outliers (e.g., outcome minimum 0%). Variability was greatest for Outcome (SD 45.9, range 0%-100%) and Patients’ rights (standard deviation [SD] 30.8, range 7.14%-100%), whereas Support services and Quality management showed minimal dispersion (interquartile range [IQR] 0, SD ≤ 17.7). Wide 95% confidence intervals (CIs), particularly for Outcome (23.3%-100%), emphasized uncertainty in these domains, given the small sample of facilities.

**Table 2:** Descriptive statistics of National Health Program (NHP) component scores across assessment domains. The table presents descriptive statistics for NHP scores (%) across various domains, including Service provisions, Patients’ rights, Inputs, Support services, Clinical services, Infection control, Quality management, and Outcome. Metrics include mean, standard error, 95% confidence intervals, median, standard deviation, variance, interquartile range (IQR), and range (minimum to maximum values). These values reflect the variability and central tendency of scores observed across urban primary health centers. Note: Confidence intervals are calculated assuming a t-distribution with *N* – 1 degrees of freedom.

Descriptive statistics									
	NHP score (%)	Service provisions	Patients' rights	Inputs	Support services	Clinical services	Infection control	Quality management	Outcome
Mean	83.2	85.7	75.9	82.5	86.6	82.9	93.8	87.5	61.7
Std. error of the mean	7.85	6.71	10.9	3.44	9.47	7.72	6.25	12.5	16.2
95% CI mean lower bound	64.6	69.9	50.2	74.4	64.2	64.7	79	57.9	23.3
95% CI mean upper bound	102	102	102	90.7	109	101	109	117	100
Median	90.4	92	78.6	84.5	96.4	91.1	100	100	83.3
Standard deviation	22.2	19	30.8	9.73	26.8	21.8	17.7	35.4	45.9
Variance	493	360	947	94.7	717	477	313	1,250	2,105
IQR	3.67	5.19	28.6	7.33	8.94	3.65	0	0	80
Range	66	58.5	92.9	31	78.6	64.6	50	100	100
Minimum	28.5	39.6	7.14	60.3	21.4	29.2	50	0	0
Maximum	94.5	98.1	100	91.4	100	93.8	100	100	100

**Figure 1:**
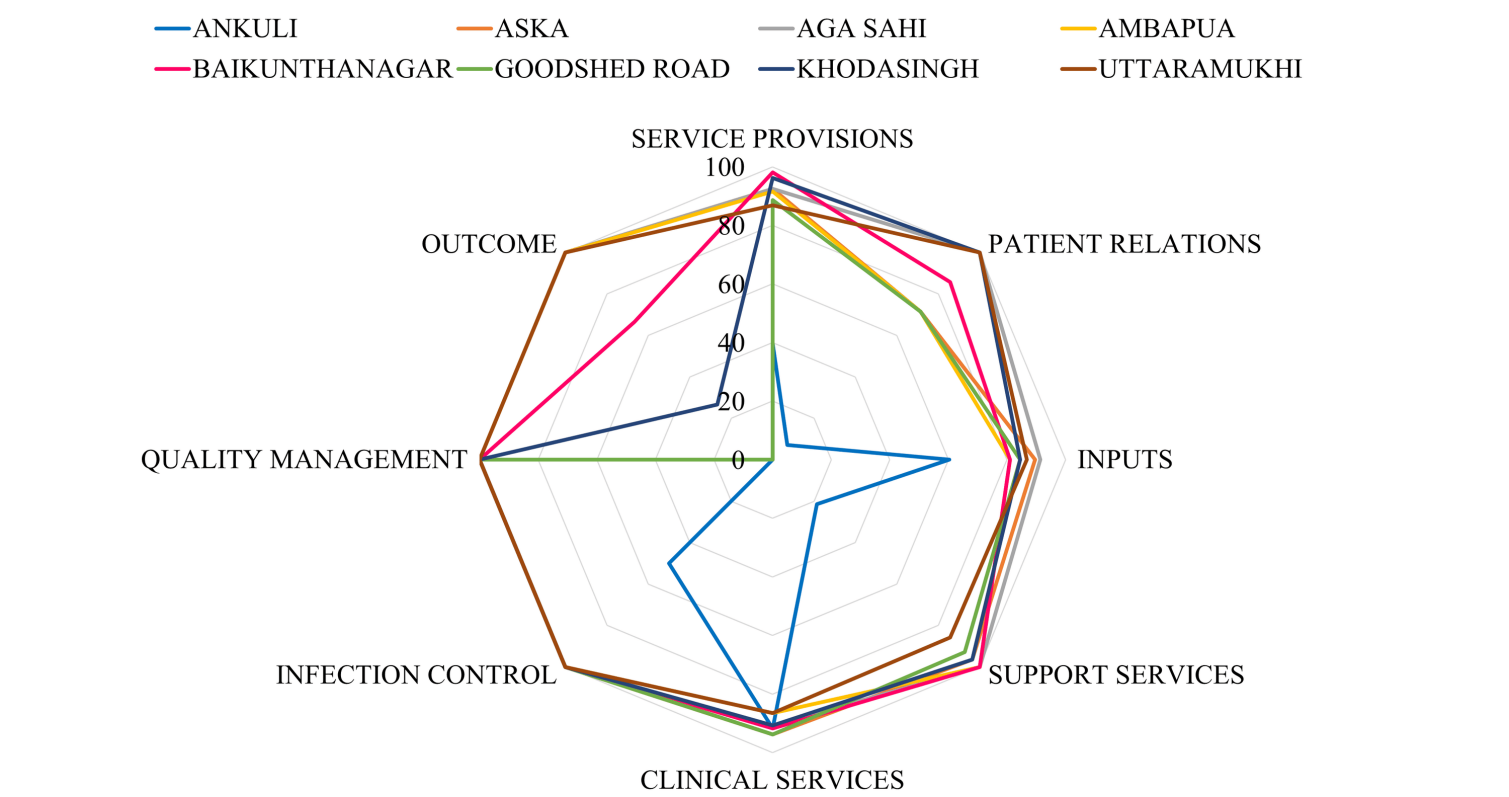
Spider diagram showing baseline assessment of quality of NHPs in UPHCs of Berhampur. The figure depicts the baseline assessment of the quality of NHPs being implemented across urban primary health centers (UPHCs) in Berhampur. It illustrates key components of healthcare delivery, including service availability, infrastructure, human resources, supply chain management, and program-specific indicators, providing a comprehensive overview of current performance levels. The authors created this image using primary data collected from the eight UPHCs in Berhampur and visualized using the MS Excel Graphs (Spider diagram). License Statement: This figure is original to the authors and is released under a CC-BY 4.0 license. Users are free to share and adapt with appropriate credit. NHP, National Health Program

The assessment of the baseline status of the quality of NHPs, ongoing in the UPHCs of Berhampur, revealed detailed insights into various components of healthcare delivery (Figure 1). Service provisions, indicative of the efficiency and effectiveness of healthcare services, vary across PHCs, with Baikunthanagar leading at 98.11%, while Ankuli lags at 39.62%. Patient relations, crucial for fostering trust and communication, range from 7.14% to 100%, with Baikunthanagar and Aga Sahi excelling. Inputs, encompassing resources and infrastructure, show disparities, with Baikunthanagar scoring 81.03%, while Ankuli scores lower at 60.34%. Support services, including administrative and ancillary support, are strong across most PHCs, with scores ranging from 85.71% to 100%. Clinical services, indicative of medical care quality, exhibit consistency, with scores ranging from 86.46% to 93.75% across PHCs. Infection control practices are robust, with most PHCs achieving 100% compliance. Quality management, essential for continuous improvement, is notably high across all PHCs. Outcomes, reflecting the effectiveness of interventions, vary, with Baikunthanagar achieving 66.67% to 100%, while Uttaramukhi scores 100%, highlighting areas for improvement and best practices across UPHCs in Berhampur. These detailed assessments offer valuable insights into the strengths and areas for improvement within each component of the UPHCs in Berhampur, facilitating targeted interventions to enhance the quality of healthcare services provided to the community.

The Kruskal-Wallis H test was conducted to assess whether there were statistically significant differences in the scores of NHP components across the eight health facilities (Table 3). Unlike the overall NHP score, which did not show significant variation, this analysis used subcomponent scores (such as Service provision, Patients’ rights, Inputs, Support services, Clinical services, Infection control, Quality management, and Outcome) as repeated observations for each facility, providing a more granular view of performance variation.

The test produced a chi-square statistic of 22.73 with 7 degrees of freedom and a corresponding *P*-value of 0.0019, indicating that the differences in scores across the facilities are statistically significant. This implies that at least one facility differs significantly from the others in its performance across the assessed programmatic components. Furthermore, the calculated effect size (epsilon squared, ε² = 0.281) suggests that approximately 28% of the variability in component scores can be attributed to differences between facilities. This represents a moderate to large effect, reinforcing that the observed differences are not only statistically significant but also meaningful in practical terms.

These findings suggest that while some facilities consistently perform well across multiple programmatic domains, others may have targeted weaknesses. Therefore, a facility-wise breakdown and focused quality improvement interventions are recommended rather than a uniform approach. This analysis underscores the value of using component-level assessments to reveal performance disparities that may be obscured when only aggregate scores are considered.

**Table 3:** Kruskal-Wallis test results for differences in NHP scores across facilities. The table presents the results of the Kruskal-Wallis test used to evaluate differences in National Health Program (NHP) scores (%) across eight facilities. The test statistic (χ²), degrees of freedom (df), *P*-value, and effect size (epsilon squared, ε²) are reported. A *P*-value of 0.0019 indicates a statistically significant difference in NHP scores between the groups. The effect size (ε² = 0.281) suggests a moderate to large effect, meaning approximately 28% of the variability in scores can be explained by the facility-level grouping.

	Kruskal-Wallis			
	*χ*²	df	*P*	*ε*²
NHP score (%)	22.73	7	0.0019	0.281

**Figure 2:**
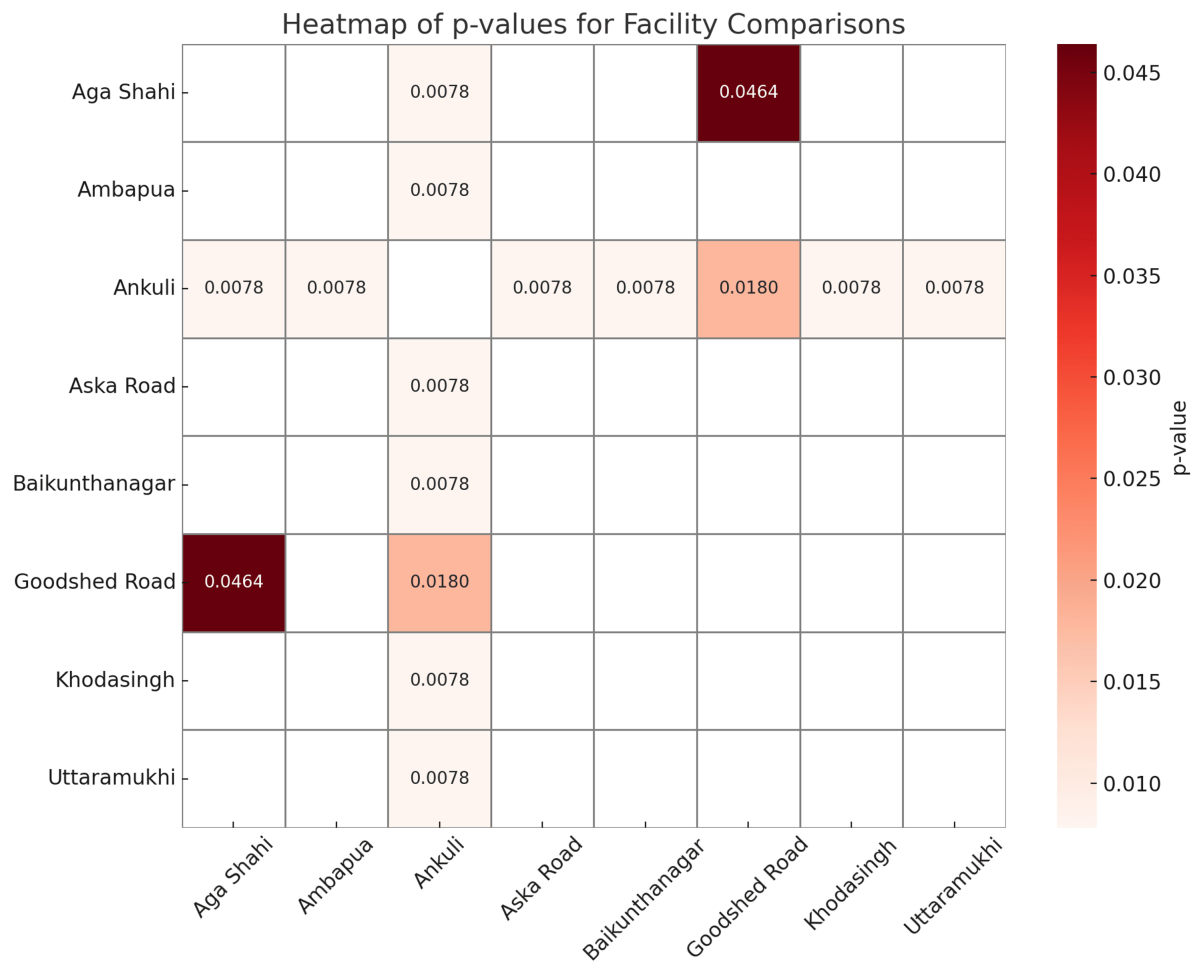
Pairwise Wilcoxon rank-sum test comparisons of NHP scores across facilities. This figure presents the results of pairwise Wilcoxon rank-sum tests comparing National Health Program (NHP) scores between individual health facilities. This non-parametric test was used to determine whether statistically significant differences existed in median NHP scores across the facilities. Adjustments for multiple comparisons were applied as appropriate.

Figure 2 is a heatmap displaying pairwise *P*-values from statistical comparisons between eight different locations: Ankuli, Aska Road, Aga Shahi, Ambapua, Baikunthanagar, Goodshed Road, Khodasingh, and Uttaramukhi. A pairwise Wilcoxon signed-rank test was used to compare component-wise NHP scores between each pair of the eight health facilities. The Wilcoxon test reinforces the evidence of heterogeneous implementation of NHPs across facilities and helps prioritize actions based on the magnitude and significance of observed disparities.

The most consistent and statistically significant findings emerged in comparisons involving Ankuli, which demonstrated markedly lower scores across nearly all programmatic dimensions. The *P*-values for comparisons between Ankuli and other facilities, such as Aska Road, Aga Shahi, Ambapua, Baikunthanagar, Khodasingh, and Uttaramukhi, were uniformly low (*P* = 0.0078). This indicates that the differences in their subcomponent scores are highly unlikely to be due to chance. In other words, Ankuli's quality of services under the NHPs is significantly inferior compared to its counterparts. These results strongly suggest that Ankuli requires immediate attention and targeted interventions to address gaps in infrastructure, service delivery, and program implementation.

Interestingly, Goodshed Road also showed statistically significant differences in a few pairwise comparisons, particularly with high-performing facilities like Aga Shahi (*P* = 0.0464). This suggests that although Goodshed Road performs reasonably well in some components, there are specific areas-especially *Outcome* and *Quality Management*, where it may be underperforming compared to top-tier centers. This nuanced insight helps direct attention not just to the worst performers but also to those with inconsistent or average patterns that might otherwise be overlooked.

In contrast, facilities such as Aska Road, Aga Shahi, Ambapua, Baikunthanagar, Khodasingh, and Uttaramukhi did not show statistically significant differences among themselves in most pairwise comparisons. Their consistently high scores across nearly all subcomponents indicate a robust and uniform standard of program implementation. This group can be considered benchmarks or *best practice* facilities within the urban primary healthcare setting studied. Such facilities might serve as model sites for peer learning, mentoring, and experience sharing.

The results highlight the importance of disaggregated, component-level analysis rather than relying solely on overall NHP scores. While aggregated data may mask intra-facility disparities, the component-wise Wilcoxon test reveals clear differences in programmatic performance. Facilities like Ankuli require focused quality improvement measures, potentially including staff training, infrastructure upgrades, and improved managerial oversight. Meanwhile, top-performing centers offer a template for scalable best practices.

## Discussion

The assessment of the baseline status of the quality of NHPs, ongoing in the UPHCs of Berhampur, sheds light on the varying levels of compliance with NQAS across different facilities. Notably, Aska Road, Aga Shahi, Ambapua, and Baikunthanagar PHCs demonstrated commendable adherence to NQAS standards, reflecting their commitment to delivering high-quality healthcare services, as evidenced by their impressive NHP scores ranging from 91.25% to 94.49%. Conversely, Ankuli PHC obtained a lower NHP score of 28.52%, indicating areas for improvement in compliance with NQAS standards. These findings resonate with existing literature, which underscores the importance of robust quality assurance mechanisms in enhancing healthcare delivery and patient outcomes. Previous studies have highlighted the positive correlation between adherence to quality standards and improved healthcare outcomes [8]. Furthermore, the detailed assessment of various components of healthcare delivery within each PHC provides valuable insights into strengths and areas for improvement. Baikunthanagar PHC's exemplary performance in service provisions, patient relations, and inputs underscores the importance of resource allocation and patient-centric care delivery models. The observed variations in compliance with NQAS among different PHCs underscore the multifaceted nature of healthcare delivery and the need for tailored interventions to address specific challenges. As evidenced by the impressive NHP scores of Aska Road, Aga Shahi, Ambapua, and Baikunthanagar PHCs, these facilities have demonstrated a strong commitment to quality assurance and service delivery. These findings resonate with prior research highlighting the positive impact of adherence to quality standards on healthcare outcomes [9]. On the other hand, Ankuli PHC's lower scores highlight potential challenges in service delivery and bring out the need for targeted interventions to address gaps in quality assurance [10]. These findings mirror national evaluations that report consistently stronger performance in management‐oriented domains (infrastructure, processes) than in patient‐centered outcomes and rights [10]. Similar studies in India have documented high adherence to structural standards but wide variation in clinical outcomes and patient‐centered measures, highlighting systemic gaps in follow‐through and service delivery [9,11]. To bridge these disparities, targeted interventions as enhanced patient feedback mechanisms and outcome monitoring are recommended, in line with WHO’s quality‐improvement frameworks [4]. Comparisons with existing literature underscore the significance of robust quality assurance frameworks in optimizing healthcare delivery. Previous studies have emphasized the importance of patient-centered care models, resource allocation, and effective communication in enhancing healthcare quality and patient satisfaction [11]. Baikunthanagar PHC's exemplary performance across various components, including service provisions, patient relations, and inputs, aligns with these principles and exemplifies best practices in healthcare delivery. Moreover, the high compliance with infection control practices across all PHCs reflects the commitment to ensuring patient safety and minimizing healthcare-associated infections [12]. While clinical services exhibit consistency across PHCs, variations in outcomes highlight the need for further investigation into factors influencing healthcare outcomes, such as patient demographics, comorbidities, and treatment adherence [13,14]. By identifying areas for improvement and best practices, this study contributes to the ongoing efforts to enhance the quality of healthcare services provided by UPHCs in Berhampur [15].

The Wilcoxon pairwise comparison and Kruskal-Wallis test (*P* = 0.0019, ε² = 0.281) confirmed that these differences were statistically significant and practically meaningful. Component-level analysis shows that Ankuli underperformed across most service domains, particularly in Patients' rights (7.14%), Outcomes (0%), and Quality management (0%), indicating systemic gaps in service delivery, user satisfaction, and process control. These findings align with the concerns raised by Rao et al. [10], who reported that primary healthcare quality in urban Indian settings is often uneven and fragmented, with resource constraints and managerial oversight being the key bottlenecks. Similarly, studies emphasized that NQAS accreditation has helped improve several public health facilities in states like Karnataka and Maharashtra, but also pointed out that poorer-performing centers struggle with leadership, training, and continuity of care issues apparent in Ankuli's performance [15]. The heatmap in Figure 2 further illustrates that Ankuli had statistically significantly lower scores in comparison with all other PHCs (*P* = 0.0078), highlighting the urgent need for focused interventions. Even in comparison with a mid-performing center like Goodshed Road (NHP score: 86.5%), the disparity is clear (*P* = 0.0180). This supports the assertion by Kumah et al. and Endalamaw et al. [8,9] that data-driven quality improvement requires not just system-wide strategies but localized corrective actions.

On the other end of the performance spectrum, facilities like Aga Shahi and Baikunthanagar achieved near-perfect compliance across several domains (e.g., Infection control and Quality management at 100%). These centers could serve as benchmarks for performance improvement in lower-scoring centers, a strategy supported by Bhattacharyya et al. [11] in their work on primary care reform in China, where high-performing centers were leveraged as models for systemic upliftment. The domain-wise descriptive statistics (Table 2) further underscore the importance of disaggregated quality monitoring. Domains such as Patients' rights (mean: 75.9%, SD: 30.8) and Outcome (mean: 61.7%, SD: 45.9) displayed the highest variability and widest confidence intervals, suggesting inconsistent attention across facilities. Duclos et al. advocate for component-specific assessments, stating that aggregate scores often mask critical service delivery failures phenomenon evident here. Moreover, while Infection control and Quality management scored consistently high across most facilities (median = 100%), this could partly reflect the checklist-driven nature of NQAS assessments, which may inadvertently favor easily auditable domains. As Chou et al. noted, quality assessments in institutional settings must balance structure, process, and outcome indicators to provide an accurate performance picture. The findings not only confirm existing disparities in primary health service delivery, as noted by Buetow and Wellingham [14], but also validate the utility of the NQAS framework in differentiating high- and low-performing centers. However, a move toward continuous quality improvement (CQI), supportive supervision, community feedback mechanisms, and resource mobilization is necessary to uplift underperforming centers like Ankuli.

The study assessing the baseline status of the quality of NHPs ongoing in the UPHCs of Berhampur exhibits several strengths. In the first place, it offers a comprehensive evaluation of healthcare delivery by employing standardized tools, particularly the NHP component of the NQAS checklist, ensuring objective measurements across multiple PHCs. The inclusion of eight UPHCs within Berhampur Municipal Corporation provides a sizable sample size for meaningful comparisons and enhances the generalizability of the findings. Moreover, the detailed analysis of various components of healthcare delivery, such as service provisions, patient relations, and clinical services, enables a nuanced understanding of the quality of care provided. Additionally, the comparison of obtained scores with established NQAS facilitates the identification of areas for improvement, guiding targeted quality enhancement efforts. However, the study is not without limitations. Its focus solely on UPHCs within Berhampur limits the generalizability of findings to other settings. While the NQAS checklist is a valuable tool for assessing healthcare quality in public health facilities, its use is subject to several limitations. Observer bias is a notable concern, as assessments rely on subjective judgments that can vary between evaluators, especially in domains like Patients' rights or Quality management. To reduce this, standardized training, independent assessments, and inter-rater reliability testing should be implemented. Additionally, the checklist's focus on structural and process indicators may overlook critical aspects like patient satisfaction, equity, and long-term outcomes. Integrating patient-reported experience measures and qualitative tools can help address this gap. The snapshot nature of NQAS assessments, reflecting a facility’s performance at a single time point, may also miss seasonal or staffing-related fluctuations. Conducting repeated assessments or continuous monitoring through digital dashboards could improve accuracy. Furthermore, facilities may prioritize checklist compliance over meaningful service improvements, highlighting the need to promote a culture of quality that values outcomes over documentation. Addressing these issues can enhance the robustness and fairness of quality evaluations in future research and practice. Despite these limitations, the study provides valuable insights into the current state of healthcare quality in UPHCs, laying the groundwork for future quality improvement initiatives. 

Based on the identified gaps in the assessment of NHP implementation across UPHCs in Berhampur, several targeted recommendations can be made for policymakers and healthcare practitioners to enhance service quality and equity. First, targeted capacity-building initiatives should be prioritized, especially in low-performing facilities such as Ankuli PHC. These should focus on training frontline health workers and facility managers in quality assurance practices, patients' rights, infection control, and data-driven decision-making. Training should also emphasize the correct application of clinical protocols and enhance understanding of the NQAS framework to ensure consistent and meaningful implementation. Second, infrastructure and resource investment are crucial. Facilities with lower scores often lack basic amenities, equipment, or human resources necessary for quality service delivery. Policymakers should allocate funds using need-based prioritization, ensuring that underperforming PHCs are equipped with adequate diagnostic tools, sanitation infrastructure, and essential drugs. Dedicated funding for maintenance and digital record-keeping systems could further streamline operations and improve accountability. Third, it is recommended to institutionalize CQI systems rather than relying solely on one-time external assessments. Facilities should be encouraged to form internal quality teams that regularly review service delivery data, engage with patient feedback, and implement Plan-Do-Study-Act (PDSA) cycles. This would foster a culture of ongoing improvement and staff ownership of quality. Fourth, patient engagement mechanisms must be strengthened. Introducing structured feedback systems-such as exit interviews, grievance redressal platforms, or periodic community satisfaction surveys-can help ensure that patient experiences are at the core of service improvement. Particular attention should be paid to capturing the voices of marginalized and vulnerable groups to promote equity in access and treatment. Finally, performance monitoring and supportive supervision must be enhanced. District and state health authorities should deploy supervisory teams trained in quality improvement to mentor PHC staff, help interpret assessment results, and support corrective actions. Creating a digital dashboard to track real-time performance metrics and sharing best practices across facilities can also drive peer learning and healthy competition. By implementing these recommendations, policymakers and practitioners can move beyond checklist-based assessments to create a resilient, responsive, and equitable primary healthcare system that consistently delivers high-quality services.

## Conclusions

This observational study provides a foundational evaluation of the quality of NHPs implemented in the UPHCs of Berhampur, offering critical insights into disparities in compliance with NQAS. The analysis revealed statistically significant variation in performance across facilities, with some PHCs, such as Aska Road, Aga Shahi, and Ambapua, demonstrating strong adherence to quality benchmarks, while others, notably Ankuli PHC, showed significant underperformance across multiple domains. These findings underscore the urgent need for targeted interventions, including capacity building, infrastructure investments, and the establishment of continuous quality improvement mechanisms. Importantly, the use of a standardized and government-endorsed tool like the NQAS checklist underscores the potential for scalability and reproducibility of this assessment approach across other urban regions in India. While limitations such as observer bias and the static nature of the evaluation warrant consideration, this study contributes meaningfully to the evidence base guiding quality improvement in primary care. For future research, a more participatory and longitudinal approach - incorporating patient feedback, outcome tracking, and periodic reassessments - would further enhance the reliability and impact of such evaluations. From a policy and practice standpoint, these findings should inform strategic resource allocation and guide supportive supervision to ensure that all PHCs, regardless of baseline performance, are equipped to deliver equitable and high-quality care.
